# Mobile Health Use by Older Individuals at Risk of Cardiovascular Disease and Type 2 Diabetes Mellitus in an Australian Cohort: Cross-sectional Survey Study

**DOI:** 10.2196/37343

**Published:** 2022-09-07

**Authors:** Vera Helen Buss, Marlien Varnfield, Mark Harris, Margo Barr

**Affiliations:** 1 Australian e-Health Research Centre Commonwealth Scientific and Industrial Research Organisation Herston Australia; 2 Centre for Primary Health Care and Equity University of New South Wales Sydney Australia

**Keywords:** cardiovascular diseases, diabetes mellitus type 2, cohort studies, telemedicine, mobile applications, mHealth, mobile health, mobile app, aging, digital health, cardiovascular, diabetes

## Abstract

**Background:**

The digital transformation has the potential to change health care toward more consumers’ involvement, for example, in the form of health-related apps which are already widely available through app stores. These could be useful in helping people understand their risk of chronic conditions and helping them to live more healthily.

**Objective:**

With this study, we assessed mobile health app use among older Australians in general and among those who were at risk of cardiovascular disease or type 2 diabetes mellitus.

**Methods:**

In this cross-sectional analysis, we used data from the second follow-up wave of the 45 and Up Study. It is a cohort study from New South Wales, Australia, with 267,153 participants aged 45 years and older that is based on a random sample from the Services Australia (formerly the Australian Government Department of Human Services) Medicare enrollment database. The 2019 follow-up questionnaire contained questions about technology and mobile health use. We further used data on prescribed drugs and hospitalizations to identify participants who already had cardiovascular disease or diabetes or who were at risk of these conditions. Our primary outcome measure was mobile health use, defined as having used a mobile health app before. We used descriptive statistics and multivariate logistic regression to answer the research questions.

**Results:**

Overall, 31,946 individuals with a median age of 69 (IQR 63-76) years had completed the follow-up questionnaire in 2019. We classified half (16,422/31,946, 51.41%) of these as being at risk of cardiovascular disease or type 2 diabetes mellitus and 38.04% (12,152/31,946) as having cardiovascular disease or type 1 or type 2 diabetes mellitus. The proportion of mobile health app users among the at-risk group was 31.46% (5166/16,422) compared to 29.16% (9314/31,946) in the total sample. Those who used mobile health apps were more likely to be female, younger, without physical disability, and with a higher income. People at risk of cardiovascular disease or type 2 diabetes mellitus were not statistically significantly more likely to use mobile health than were people without risk (odds ratio 1.06, 95% CI 0.97-1.16; *P*=.18; adjusted for age, sex, income, and physical disability).

**Conclusions:**

People at risk of cardiovascular disease or type 2 diabetes mellitus were not more likely to use mobile health apps than were people without risk. Those who used mobile health apps were less likely to be male, older, with a physical disability, and with a lower income. From the results, we concluded that aspects of equity must be considered when implementing a mobile health intervention to reach all those that can potentially benefit from it.

## Introduction

Chronic conditions pose a great burden to health systems [1]. One of the main drivers for the high prevalence of chronic diseases such as cardiovascular disease (CVD) and type 2 diabetes mellitus (T2DM) is an unhealthy lifestyle [2]. This includes smoking, high alcohol consumption, a lack of physical activity, and a poor diet [2]. Health promotion campaigns aim at increased awareness for lifestyle-related risk factors and support for risk reduction [3]. Digital applications, such as mobile health (mHealth) apps for smartphones, offer an excellent opportunity to make such health promotion interventions available to as many people as possible [3]. A report by the IQVIA Institute stated that in 2021, over 350,000 health-related apps were available through app stores [4]. According to the report, 90,000 new health-related apps appeared on the market in 2020 alone [4]. The mHealth space is not limited to apps; other products include smart watches and activity trackers, commonly called wearables [5].

Meskó et al [6] called digital health the cultural transformation of traditional health care. In a recent position paper by the European Society of Cardiology Working Group on e-cardiology [5], the authors mentioned that the advantages of wearables include the democratization of health data, the potential for earlier detection of risk factors and disease states, and increased health awareness. The authors also noted that while older and high-risk individuals would likely benefit the most, mHealth technology is currently used the most by the younger, healthy generation with higher income [5]. More generally, Spoth et al [7] reported that those who are most in need of evidence-based prevention and health promotion interventions often do not use them, in many cases due to lack of access. To understand the possible uptake of an app-based preventive intervention for CVD and T2DM that we are currently developing, we examined mHealth use among older Australians. Previous research has reported on mHealth uptake in other countries. For example, Shan et al [8], Robbins et al [9], Rising et al [10], and Carroll et al [11] conducted studies using US data; Pare et al [12] analyzed Canadian data; and Xi et al [13] investigated mHealth uptake in Chinese adults. However, so far there has not been a larger study looking at mHealth use in the Australian setting. For this purpose, we analyzed data on mHealth use collected in 2019 from the second follow-up wave of the 45 and Up Study which is a large cohort study of Australians who were aged 45 years and older at baseline between 2006 and 2009 [14,15]. With this study, we also aimed to identify any distinguishing features between those who used mHealth and those who did not. Our specific research questions were as follows: What was the proportion of mHealth users overall and among those who were at risk of CVD or T2DM? How did mHealth users differ from non-mHealth users for people at risk of CVD or T2DM? Were those at risk of CVD or T2DM more likely to use mHealth than those without risk?

## Methods

### Ethics Approval

This cross-sectional study was based on survey data from the Sax Institute’s 45 and Up Study. This is a large cohort including 267,153 participants aged 45 years and older who reside in New South Wales (NSW), Australia [16]. The conduct of the 45 and Up Study was approved by the University of New South Wales Human Research Ethics Committee. The survey data were linked to data from the Pharmaceutical Benefits Scheme (PBS; data on prescribed drugs) and the NSW Admitted Patient Data Collection (APDC; hospitalization data). This study has been approved by the NSW Population & Health Services Research Ethics Committee (approval #HREC/16/CIPHS/14) and the Commonwealth Scientific and Industrial Research Organisation (CSIRO) Health and Medical Human Research Ethics Committee (approval #2021_018_RR). All cohort participants provided free and informed consent.

### Data Sources

The recruitment for the 45 and Up Study took place between January 2006 and December 2009 and was based on a random sample from the Services Australia (formerly the Australian Government Department of Human Services) Medicare enrollment database, with oversampling in people aged 80 years and over and residents of rural and remote areas [16]. About 18% of those who were contacted consented to take part in the study which represents 11% of the NSW population aged 45 years and older [14]. A detailed description of the cohort and the study methods was published by the 45 and Up Study collaborators [14,15]. For our analyses, we used data from the second wave of follow-up collected between 2018 and 2020, specifically, the 2019 follow-up survey, which included questions about participants’ technology and mHealth use [17]. The survey was sent to 68,349 participants, of whom 31,965 responded (46.77%) [16]. In general, the questionnaires contained questions on lifestyle, medical history, family history of chronic conditions, socioeconomic status, and geographic factors [17]. The Sax Institute linked the survey data to the PBS data deterministically using a unique identifier [16]. Additionally, the Centre for Health Record Linkage linked these data to the APDC data using probabilistic techniques [18].

### Subgroups

We first identified participants with CVD or diabetes (type 1 or 2, not including gestational diabetes) at the time of the follow-up survey and then identified those who were at risk of the conditions. These methods were similar to the methods described by Joshy et al [19] for the classification of CVD and by Comino et al [20] for the classification of diabetes in the 45 and Up Study. Individuals were classified based on information from the survey, from the APDC data, and from the PBS data. In the survey, participants were asked if they had CVD or diabetes (“Has a doctor ever told you that you have:” heart failure, atrial fibrillation, other heart disease, stroke, OR diabetes—type 1, type 2 or unsure) and if they took medication for these conditions (corresponding medication listed on the questionnaire: Cardizem/Vasocardol, warfarin, OR Diabex/Diaformin). From the APDC data, the diagnoses were classified based on related hospital admissions before the date of the survey. We searched for relevant International Classification of Diseases version 10 Australian Modification (ICD-10-AM) diagnosis codes in any of the 55 diagnostic fields or relevant Australian Classification of Health Interventions procedure codes in any of the 50 procedure code fields. These codes were based on methods described by the Australian Institute of Health and Welfare (AIHW) [21-24]. From the PBS data, people were classified with CVD or diabetes if they took medication with these indications in the past 12 months [25,26]. For CVD, the only drugs considered were those that are solely indicated for CVD. To identify a CVD diagnosis, we did not consider drugs for hypertension or dyslipidemia. People with hypertension or dyslipidemia may be at risk of CVD but have not yet developed the condition. Additionally, people may take antihypertensive drugs for other reasons than hypertension. Therefore, if people took antihypertensive or lipid-modifying drugs but did not have a diagnosis of CVD (ie, reported in the 45 and Up Study data, the APDC data, or due to taking a drug with CVD as the only indication), we did not classify them as having the condition.

For CVD, being at risk was classified as taking antihypertensive medication, lipid-modifying medication, or low-dose aspirin; reporting blood-clotting problems or hypertension; being obese (≥30 kg/m^2^); or having a family history of CVD (parents or siblings) and not already having CVD. For T2DM, someone was classified to be at risk if they had had gestational diabetes, were obese (≥30 kg/m^2^), or had a family history of T2DM (parents or siblings) but were free of type 1 or T2DM. We gathered the information to identify at-risk populations from the survey and the PBS data. Women were classified as having had gestational diabetes if they self-reported having had the condition or if they had received a diabetes diagnosis before the date of their last delivery and if there was no evidence of diabetes medication in their records for the 12 months before filling out the follow-up survey.

### Technology and mHealth Use

The primary outcome was mHealth use. We defined mHealth use through the following survey question: “How often do you use apps on your mobile phone or tablet to track the following?” The answer options were “never,” “less than once a month,” “at least once a month,” “at least once a week,” or “every day.” If any of the options were selected with “less than once a month” or more frequently, the person was classified as a mHealth user (options included the following: activity or fitness, vital signs, nutrition or weight, mood or well-being, sleep, medications). This meant that if “never” or none of the options was selected, the participant was considered a nonuser. Table 1 summarizes the technology-related questions from the 45 and Up Study questionnaire that we used for the analysis [17]. We classified all those who did not select yes for the questions about device use (computer or laptop, tablet, smartphone, fitness tracker, and smart watch) as not using them. We categorized everyone as app users who did not select “I don’t use apps/don’t know what apps are.” We dichotomized the variable to app download by putting app nonusers in one group with those who selected that they had not downloaded any apps yet and the remaining in the other group.

**Table 1:** Technology-related questions from the 45 and Up Study questionnaire 2019 that were used in the analysis.

Question	Answer options
Do you use a computer or mobile device (eg, phone with a touch screen, tablet, or smart watch) regularly?	Yes/no
If YES, which of the following devices do you use regularly? Apple desktop or laptop computer (eg, iMac, MacBook)/Windows desktop or laptop computer/Apple tablet (iPad)/other tablet (eg, Samsung, Microsoft Surface, Lenovo)/Apple phone with a touch screen (iPhone)/Android phone with a touch screen (eg, Samsung, Huawei, Google)/other phone with a touch screen/Apple Watch/other smart watch/fitness tracker (eg, Fitbit, Garmin)	Yes/no/unsure
If you use applications (apps) on a mobile phone or tablet, how many have you ever downloaded yourself? (choose one only)	I don’t use apps/don’t know what apps are/none/1-5 /6 or more
How often do you use apps on your mobile phone or tablet to track the following: activity or fitness (eg, number of steps, exercise)/vital signs (eg, heart rate, blood pressure, breathing)/nutrition or weight/mood or well-being/sleep/medications (eg, reminders, alerts)	Never/less than once a month/at least once a month/at least once a week/every day

### Other Measures

Other variables of interest included age, sex, income, lifestyle (smoking, alcohol, fruit or vegetables, physical activity), and physical disability. We categorized income based on the Organization for Economic Cooperation and Development (OECD) report [27] into low (less than Aus $30,000 per year [US $20,580]), middle (Aus $30,000-89,999 per year [US $20,580-61,740]), and high income (Aus $90,000 or more per year [US $61,741 or more]). For smoking, we created three categories: never, past, and current smokers. We classified physical disability through survey questions about illness or disability restricting physical activity (“Is there anything that stops you from participating in physical activity?”—ill-health; “Do you regularly need help with daily tasks because of long-term illness or disability?”—yes; “Does your health now limit you in any of the following activities?”—yes selected for any of the options). We dichotomized all other lifestyle-related variables depending on if participants met the guideline recommendations (for alcohol: maximum 10 standard drinks per week [28]; for fruits and vegetables: at least 2 servings of fruits and 5 servings of vegetables per day [29]; for physical activity: at least 150 minutes of physical activity per week with vigorous physical activity counting double [30]). For fruits and vegetables, we set servings per day to 0 if the participants selected that they did not eat any fruits or vegetables, respectively. Otherwise, we did not impute any missing values. We reported on the percentage of missingness for age, sex, BMI, smoking status, alcohol consumption, fruit and vegetable intake, physical activity, income, and physical disability. For the remaining variables, we did not report on missing values because the way we categorized these variables did not result in any missing values.

### Statistical Analysis

We used descriptive statistics to describe the demographics of the overall population and the various subgroups (with CVD or diabetes; at risk of CVD or T2DM; free of CVD, T2DM, and their risk factors) as well as their technology and mHealth use. Further, we used the *t* test and the chi-square test to check for differences between mHealth users and nonusers among those at risk of CVD or T2DM. We defined statistical significance at a level of 5%. We built multivariable logistic regression models for the total sample and those at risk of CVD or T2DM to assess the influence of age, sex, physical disability, and income (predictor variables) on mHealth use (outcome variable). From these models, we calculated adjusted odds ratios (ORs) and their 95% CIs. Further, we computed the adjusted OR of mHealth use for someone with CVD or T2DM risk compared to someone free of the condition and not at risk. We conducted the analyses in RStudio (version 1.2.5042) using the programming language R (version 4.0.0; R Foundation for Statistical Computing) within the Secured Unified Research Environment (SURE) provided by the Sax Institute [31].

## Results

**Figure 1:**
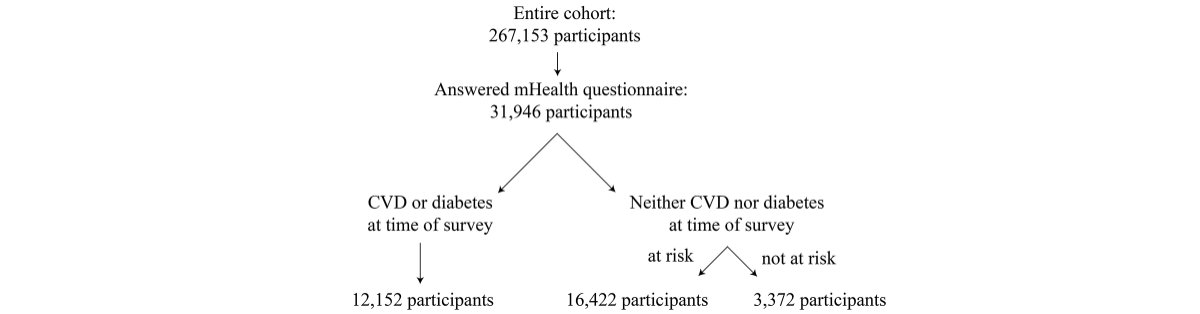
Flowchart of study participants. CVD: cardiovascular disease; mHealth: mobile health.

In total, 31,946 individuals completed the follow-up questionnaire in 2019 that contained the mHealth questions. From these 31,946 participants, 12,152 (38.04%) participants had CVD or diabetes at the time of the follow-up survey, 16,422 (51.41%) participants were categorized as at risk of the conditions, and 3372 (10.56%) participants were categorized as not at risk (Figure 1). The variable with the highest proportion of missing values was fruit and vegetable intake with 7.66% (2447/31,946; Table 2).

Table 3 compares the characteristics of the total sample and the various subgroups, including information on device ownership and app use. The group with CVD or diabetes included older people, more men, more people with physical disabilities, more people who were obese, and more exsmokers than did the at-risk group. The difference for these variables was even greater when compared to the group without CVD, diabetes, and risk factors. Device ownership was higher in people without CVD and diabetes. Overall, 75.93% (24,256/31,946) of the total sample stated that they owned a smartphone and 29.16% (9314/31,946) of the total sample stated that they had used a health app before. The highest proportion was in the group without conditions and risk factors (1102/3372, 32.68%), followed by the at-risk group (5166/16,422, 31.46%), and last the group with CVD or diabetes (3046/12,152, 25.07%).

**Table 2:** Proportion of missing values for each variable of interest (N=31,946).

Variable	Missing, n (%)
Age	0 (0)
Sex	0 (0)
BMI	1528 (4.78)
Smoking status	282 (0.88)
Alcohol consumption	697 (2.18)
Fruit and vegetable intake	2447 (7.66)
Physical activity	964 (2.96)
Income	1303 (4.08)
Physical disability	1745 (5.46)

**Table 3:** Demographic characteristics of all participants and various subgroups.

Characteristics			Total sample		With CVD^a^ or diabetes		At risk of CVD or T2DM^b^		Free of CVD and diabetes and not at risk
Subgroup, n (% of total sample)			31,946 (100)		12,152 (38.04)		16,422 (51.41)		3372 (10.56)
Age, median (IQR)			69 (63-76)		73 (67-79)		67 (62-3)		65 (61-71)
Female, n (%)			16,462 (51.53)		5181 (42.63)		9517 (57.95)		1764 (52.31)
Physical disability, n (%)			14,963 (49.54)		7598 (65.60)		6433 (41.56)		932 (29.68)
**Weight, n (%)**									
	Normal	10,677 (35.10)		3302 (28.69)		5558 (35.39)		1817 (56.69)	
	Overweight	11,851 (38.96)		4475 (38.88)		5988 (38.13)		1388 (43.31)	
	Obese	7890 (25.94)		3733 (32.43)		4157 (26.47)		0 (0)	
**Smoking status, n (%)**									
	Never	19,259 (60.82)		6801 (56.55)		10,288 (63.15)		2170 (64.85)	
	Past	11,508 (36.34)		4924 (40.94)		5528 (33.93)		1056 (31.56)	
	Current	897 (2.83)		302 (2.51)		475 (2.92)		120 (3.59)	
**Meeting guideline recommendations, n (%)**									
	Alcohol	24,971 (79.9)		9621 (81.62)		12,760 (79.02)		2590 (78.13)	
	Fruits and vegetables	7039 (23.9)		2572 (23.26)		3744 (24.40)		723 (23.35)	
	Physical activity	24,025 (77.5)		8111 (69.26)		13,159 (81.97)		2755 (85.61)	
**Income, n (%)**									
	Low	6377 (20.8)		3128 (27.16)		2770 (17.44)		479 (14.74)	
	Middle	12,789 (41.7)		4705 (40.86)		6732 (42.40)		1352 (41.61)	
	High	6486 (21.2)		1810 (15.72)		3769 (23.74)		907 (27.92)	
	Prefer not to say	4991 (16.3)		1872 (16.26)		2608 (16.42)		511 (15.73)	
**Device ownership, n (%)**									
	Laptop or computer	22,610 (70.78)		7891 (64.94)		12,160 (74.05)		2559 (75.89)	
	Tablet	15,368 (48.11)		5209 (42.87)		8506 (51.80)		1653 (49.02)	
	Smartphone	24,256 (75.93)		8198 (67.46)		13,319 (81.10)		2739 (81.23)	
	Fitness tracker	3523 (11.03)		1118 (9.20)		1999 (12.17)		406 (12.04)	
	Smart watch	1506 (4.71)		513 (4.22)		811 (4.94)		182 (5.40)	
**App use, n (%)**									
	Any app	26,434 (82.75)		9468 (77.91)		14,050 (85.56)		2916 (86.48)	
	Downloading apps	22,336 (69.92)		7763 (63.88)		12,042 (73.33)		2540 (75.33)	
	Health apps	9314 (29.16)		3046 (25.07)		5166 (31.46)		1102 (32.68)	

^a^CVD: cardiovascular disease.

^b^T2DM: type 2 diabetes mellitus.

Of those who used mHealth, physical activity was the most tracked feature (Table 4). Overall, 24.73% of participants (7900/31,946) had tracked their physical activity levels with an app before. Among those who were at risk of CVD or T2DM, the proportion was slightly higher at 27.20% (4467/16,422). The second most-tracked feature was vital signs (total: 7315/31,946, 22.90%; at risk: 4168/16,422, 25.38%). All other features were tracked by less than 10% of the sample. Among those who stated that they tracked physical activity, most did so daily (total: 3600/31,946, 11.27%; at risk: 2032/16,422, 12.37%). The same tendency was observed for tracking medication, sleep, and vital signs. For mood and weight or diet, those who stated that they tracked the feature were mostly reporting a frequency of less than once a month.

We observed statistically significant differences between mHealth users and nonusers among the at-risk population for the variables sex, hypertension, gestational diabetes, family history, physical disability, weight, smoking status, meeting physical activity guideline recommendations, and owning technical devices (Table 5).

**Figure 2:**
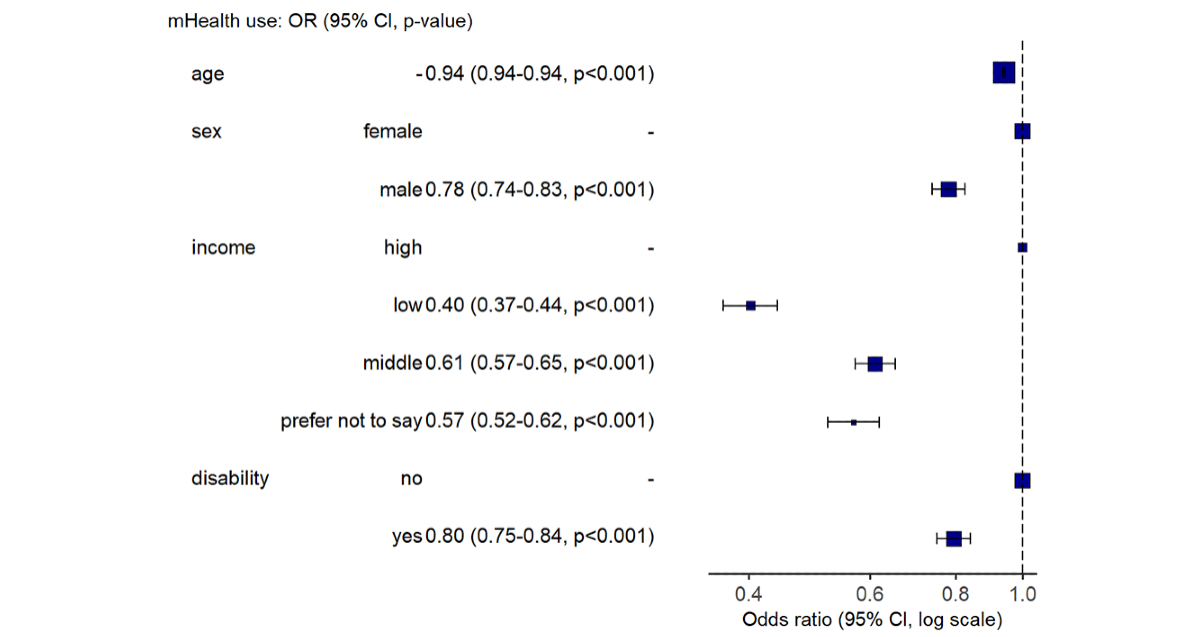
Forest plot with adjusted odds ratios for using mHealth in the entire cohort. mHealth: mobile health.

**Figure 3:**
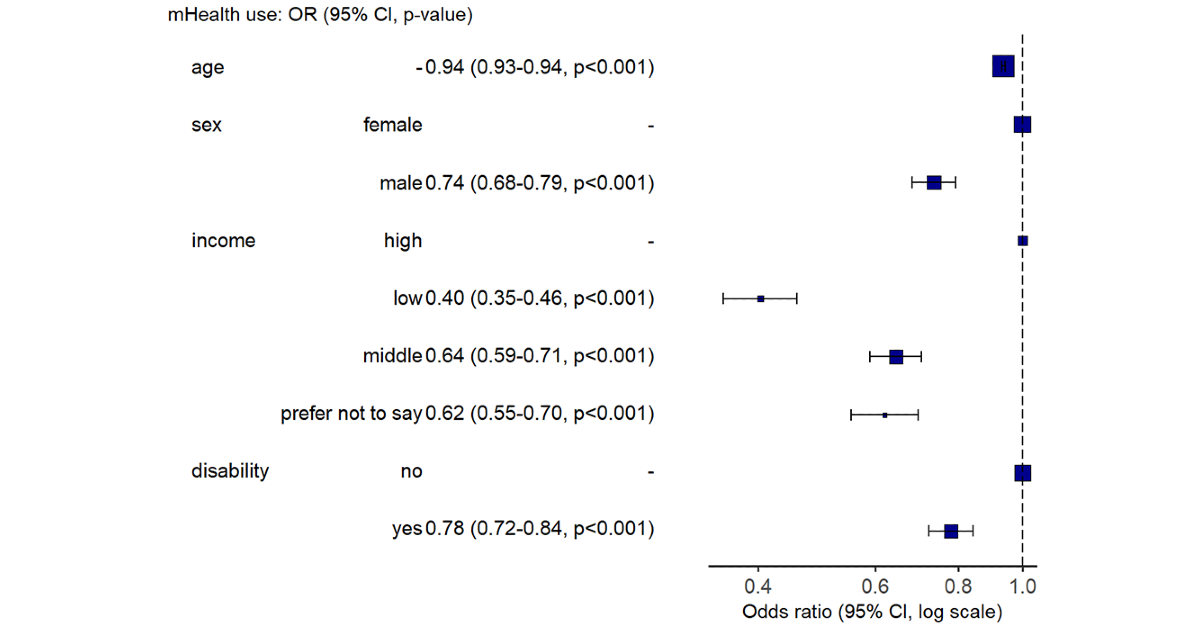
Forest plot with adjusted odds ratios for using mHealth in those at risk of cardiovascular disease or type 2 diabetes mellitus. mHealth: mobile health.

When adjusted for other demographic factors, younger participants, women, people without physical disability, and people with higher income were more likely to use mHealth (Figure 2 for all participants and Figure 3 for the at-risk population). The adjusted OR of using mHealth for someone who was at risk of CVD or T2DM compared to someone who was not at risk and free of both conditions was 1.06 (95% CI 0.97-1.16; *P*=.18; adjusted for age, sex, income, and physical disability).

**Table 4:** Frequency of mHealth use (apps with health-related tracking features) overall and among the subgroups.

Sample by frequency of tracking		Physical activity, n (%)^a^	Medication, n (%)^a^	Mood, n (%)^a^		Weight or diet, n (%)^a^		Sleep, n (%)^a^	Vital signs, n (%)^a^
**Total**									
	Never	24,046 (75.27)	30,552 (95.64)		31,133 (97.46)		29,344 (91.86)	29,418 (92.09)	24,631 (77.10)
	<1/month	1314 (4.11)	302 (0.95)		389 (1.22)		828 (2.59)	485 (1.52)	1254 (3.93)
	Monthly	790 (2.47)	217 (0.68)		130 (0.41)		460 (1.44)	307 (0.96)	731 (2.29)
	Weekly	2,196 (6.87)	247 (0.77)		156 (0.49)		716 (2.24)	572 (1.79)	1983 (6.21)
	Daily	3,600 (11.27)	628 (1.97)		138 (0.43)		598 (1.87)	1,164 (3.64)	3347 (10.48)
**With CVD^b^ or diabetes**									
	Never	9696 (79.80)	11,480 (94.47)		11,906 (97.98)		11,297 (92.96)	11,361 (93.49)	9921 (81.64)
	<1/month	404 (3.32)	124 (1.02)		105 (0.86)		243 (2.00)	132 (1.09)	377 (3.10)
	Monthly	240 (1.97)	96 (0.79)		45 (0.37)		167 (1.37)	91 (0.75)	217 (1.79)
	Weekly	679 (5.59)	117 (0.96)		47 (0.39)		228 (1.87)	183 (1.51)	592 (4.87)
	Daily	1,133 (9.32)	335 (2.76)		49 (0.40)		217 (1.79)	385 (3.17)	1045 (8.60)
**At risk of CVD or T2DM^c^**									
	Never	11,955 (72.80)	15,806 (96.25)		15,949 (97.12)		14,940 (90.98)	14,966 (91.13)	12,254 (74.62)
	<1/month	739 (4.50)	152 (0.93)		236 (1.44)		495 (3.01)	294 (1.79)	712 (4.34)
	Monthly	459 (2.80)	103 (0.63)		68 (0.41)		246 (1.50)	182 (1.11)	428 (2.61)
	Weekly	1237 (7.53)	106 (0.64)		90 (0.55)		411 (2.50)	313 (1.91)	1135 (6.91)
	Daily	2032 (12.37)	255 (1.55)		79 (0.48)		330 (2.01)	667 (4.06)	1893 (11.53)
**Free of CVD and diabetes and not at risk**									
	Never	2395 (71.03)	3266 (96.86)		3278 (97.21)		3107 (92.14)	3091 (91.67)	2456 (72.84)
	<1/month	171 (5.07)	26 (0.77)		48 (1.42)		90 (2.67)	59 (1.75)	165 (4.89)
	Monthly	91 (2.70)	18 (0.53)		17 (0.50)		47 (1.39)	34 (1.01)	86 (2.55)
	Weekly	280 (8.30)	24 (0.71)		19 (0.56)		77 (2.28)	76 (2.25)	256 (7.59)
	Daily	435 (12.90)	38 (1.13)		10 (0.30)		51 (1.51)	112 (3.32)	409 (12.13)

^a^Percentages may not total 100% due to rounding.

^b^CVD: cardiovascular disease.

^c^T2DM: type 2 diabetes mellitus.

**Table 5:** Differences between participants at risk of CVD and/or T2DM who use mHealth and who do not.

Characteristics			At risk of CVD^a^ or T2DM^b^						
			mHealth^c^ users, n (%)^d^		Nonusers, n (%)^d^	Chi-square (*df*)^e^			*P* value^e^
Sample size			5166 (31.46)		11,256 (68.54)	N/A^f^			N/A
Age, median (IQR) ^g^			64 (60-69)		69 (63-75)	N/A			.47
Female			3177 (61.50)		6340 (56.33)	38.7 (1)			<.001
Hypertension			2199 (42.57)		5355 (47.57)	35.5 (1)			<.001
Dyslipidemia			1539 (29.79)		3462 (30.76)	1.5 (1)			.22
Gestational diabetes, n (% of women)			46 (1.45)		56 (0.88)	5.8 (1)			.02
Family history of CVD			3564 (68.99)		7488 (66.52)	9.7 (1)			.002
Family history of diabetes			1421 (27.51)		2653 (23.57)	29.2 (1)			<.001
Physical disability			1611 (32.80)		4822 (45.63)	226.5 (1)			<.001
**Weight**							9.8 (2)		.01
	Normal	1690 (33.73)		3868 (36.18)					
	Overweight	1981 (39.53)		4007 (37.48)					
	Obese	1340 (26.74)		2817 (26.35)					
**Smoking status**							38.4 (2)		<.001
	Never	3225 (62.71)		7063 (63.36)					
	Past	1826 (35.50)		3702 (33.21)					
	Current	92 (1.79)		383 (3.44)					
**Following guideline recommendations**									
	Alcohol	4058 (79.29)		8702 (78.90)		0.3 (1)		.59	
	Fruits and vegetables	1225 (24.85)		2519 (24.18)		0.8 (1)		.38	
	Physical activity	4505 (88.59)		8654 (78.90)		220.2 (1)		<.001	
**Income**							626.4 (3)		<.001
	Low	500 (9.89)		2270 (20.98)					
	Middle	2,034 (40.22)		4698 (43.41)					
	High	1750 (34.61)		2019 (18.66)					
	Prefer not to say	773 (15.29)		1835 (16.96)					
**Device ownership**									
	Laptop or computer	4394 (85.06)		7766 (68.99)		474.5 (1)		<.001	
	Tablet	3510 (67.94)		4996 (44.39)		786.2 (1)		<.001	
	Smartphone	4982 (96.44)		8337 (74.07)		1154.9 (1)		<.001	
	Fitness tracker	1694 (32.79)		305 (2.71)		2994.3 (1)		<.001	
	Smart watch	686 (13.28)		125 (1.11)		1114.3 (1)		<.001	

^a^CVD: cardiovascular disease.

^b^T2DM: type 2 diabetes mellitus.

^c^mHealth: mobile health.

^d^Age is presented as median (IQR).

^e^For age: Wilcoxon rank-sum test with continuity correction; for all other variables: Pearson chi-square test with Yates continuity correction.

^f^N/A: not applicable.

^g^W=3.

## Discussion

### Principal Findings

The overall aim of this analysis was to understand how older Australians in general and particularly those at risk of CVD or T2DM use mHealth. To our knowledge, this is the first study of this kind in Australia. Among the at-risk population, the proportion of mHealth users was slightly higher than that of the general proportion. The multivariable logistic regression analysis showed that women, younger people, individuals without disabilities, and higher earners had higher odds of using mHealth. Among the mHealth users, there were fewer smokers and fewer people with hypertension or physical disability. On the other hand, among those who did not use mHealth, fewer people were overweight and fewer reported a family history of CVD or T2DM. According to our results, people at risk of CVD or T2DM do not have higher odds of using mHealth than do those without risk.

### Comparison to Other Work

The results largely corresponded to the results of other researchers. Shan et al [8] compared mHealth use among US Americans with CVD or at risk with those without based on data from 2018. They concluded that those who had or were at risk of CVD were less likely to use mHealth than were the rest of the study population in an unadjusted comparison. However, when adjusted for age, race, education, household income, health insurance, and urban or rural location, the OR of having a health app on the smartphone was 1.24 (95% CI 0.85-1.81; *P*=.26) for women with CVD or at risk of CVD and 1.12 (95% CI 0.68-1.84; *P*=.65) for men with CVD or at risk of CVD compared to women and men with no history or risk factors of CVD. Seifert and Vandelanotte [32] reported on the use of wearables and mHealth apps among Swiss adults aged 65 years and older based on data from 2019. Of their 1149 participants, 43.1% owned a tablet, 68.7% owned a smartphone, 7.6% owned a fitness tracker, 3.3% owned a smart watch, and 22.9% used mHealth apps. Bhuyan et al [33] analyzed 2014 data from US American adults on mHealth use and health-oriented behavior. They found that 35.9% of the smartphone or tablet owners had mHealth apps installed. The proportion of those who had mHealth apps was much lower among those aged 55 to 64 years (10.6%) and those aged 65 years and over (4.7%). Robbins et al [9] analyzed 2015 survey data on mHealth use among US American adults who owned a mobile phone. Among the participants, daily mHealth use was more common in individuals without diseases (21.3%) than in people with high blood pressure (2.7%), with obesity (13.1%), with diabetes (12.3%), with depression (12.0%), or with high cholesterol (16.6%). Robbins et al [9] concluded that their findings suggest that those most likely to benefit from mHealth are least likely to use it.

### Limitations

Only 18% of those who were invited to participate in the 45 and Up Study took the baseline survey [14]. This is comparable to other large international cohort studies [34-36]. Mealing et al [37] conducted an analysis in which they compared exposure-outcome relationships in the 45 and Up Study to the NSW Population Health Survey which had a response rate of about 60% [38]. Both are based on the same population, and the analysis showed that the results from both cohorts can be generalized. Additionally, some participants were lost to follow-up, which might also raise concerns about generalizability. Wang et al [39] built a logistic regression model to assess the influence of nonresponse in the first follow-up survey of the 45 and Up Study. In their conclusion, they reported that it did not lead to substantial bias and essentially did not change the interpretation of the results [39]. Further limitations were that the data set had missing values, especially concerning fruit and vegetable intake, and was based on self-report. Ng et al [40] considered the bias through self-reported weight and height in the 45 and Up Study. They stated that the resulting BMI values were logical but underestimated being overweight or obese [40]. There is also a risk of misclassification of disease diagnosis or risk. To minimize the risk, we applied methods that have been described by the AIHW [22-24] and by other researchers who worked with the 45 and Up Study [19,20]. In our analysis, we did not classify diabetes as a risk factor for CVD because we categorized people with CVD or T2DM as a separate subgroup. There is a small risk of wrongly classifying people as at risk of CVD because they took antihypertensive drugs for an indication unrelated to an increased CVD risk.

### Implications

The availability of mHealth keeps increasing, and there is little doubt that it can positively impact health promotion, risk awareness, early detection, and, in general, engagement with one’s health and well-being. However, this study and other research have shown that certain demographics are more likely to use it than are others. In our study, younger females without disabilities and with higher income had the highest odds of mHealth use. As we are in the process of developing an app-based intervention for CVD and T2DM prevention, we are interested in finding strategies that would facilitate optimal uptake by those who would be most likely to benefit from the intervention. Our findings indicate that health-related apps were less used by people with a physical disability. We think that people with disabilities would likely benefit from more personalized interventions that take into account their disability. Moreover, the results showed that it will be important to consider equity issues to ensure that people with low income and older people will not be left out. Other researchers have drawn similar conclusions and proposed solutions. For example, Foley et al [41] used a mixed methods study to explore the access, use, and benefits of digital health services in Australia and identified trust as a key factor for digital health services use. They explained that recommendations by health professionals improved trust in digital health services, which could lead to increased uptake [41]. Cheng et al [42] concluded in their systematic review that the level of electronic health literacy was often overlooked when designing such interventions, leading to a digital divide with socially disadvantaged groups being left behind. Therefore, they demanded that electronic health literacy levels need to be recognized when developing interventions [42]. In their review, Borg et al [43] identified attitudes, skills, and access as barriers to digital inclusion, and social support, education, and inclusive design as enablers. The authors highlighted the importance of user-focused and collaborative designs to ensure digital inclusion [43].

### Conclusions

Despite most people at risk of CVD or T2DM owning a smartphone, only about a third had ever used mHealth apps. There was no difference in mHealth use between people at risk of CVD or T2DM and those not at risk. People who used mHealth apps were less likely to be male, older, with physical disability, and with a lower income. This shows that it is important to consider equity issues when implementing a mHealth intervention. For example, low income or older age should not prevent people from participating in the intervention, and, therefore, these factors should be considered when developing an implementation strategy.

## Abbreviations

AIHW: Australian Institute of Health and Welfare
APDC: Admitted Patient Data Collection
CSIRO: Commonwealth Scientific and Industrial Research Organisation
CVD: cardiovascular disease
ICD-10-AM: International Classification of Diseases version 10 Australian Modification
mHealth: mobile health
NSW: New South Wales
OECD: Organization for Economic Cooperation and Development
OR: odds ratio
PBS: Pharmaceutical Benefits Scheme
SURE: Secure Unified Research Environment
T2DM: type 2 diabetes mellitus

## References

[1] Bertram MY, Sweeny K, Lauer JA, Chisholm D, Sheehan P, Rasmussen B, Upreti SR, Dixit LP, George K, Deane S (2018). Investing in non-communicable diseases: an estimation of the return on investment for prevention and treatment services. Lancet.

[2] GBD 2019 Risk Factors Collaborators (2020). Global burden of 87 risk factors in 204 countries and territories, 1990-2019: a systematic analysis for the Global Burden of Disease Study 2019. Lancet.

[3] Atun R, Jaffar S, Nishtar S, Knaul FM, Barreto ML, Nyirenda M, Banatvala N, Piot P (2013). Improving responsiveness of health systems to non-communicable diseases. Lancet.

[4] (2021). Digital health trends 2021: Innovation, evidence, regulation, and adoption. IQVIA Institute for Human Data Science.

[5] Jensen M, Treskes R, Caiani E, Casado-Arroyo R, Cowie M, Dilaveris P, European Association of Preventive Cardiology, European Heart Rhythm Association, Association of Cardiovascular Nursing and Allied Professionals (2021). ESC working group on e-cardiology position paper: Use of commercially available wearable technology for heart rate and activity tracking in primary and secondary cardiovascular prevention, in collaboration with the European Heart Rhythm Association, European Association of Preventive Cardiology, Association of Cardiovascular Nursing and Allied Professionals, Patient Forum, and the Digital Health Committee. Eur Heart J Digit Health.

[6] Meskó B, Drobni Z, Gergely B, Győrffy Z, Bényei (2017). Digital health is a cultural transformation of traditional healthcare. Mhealth.

[7] Spoth R, Rohrbach LA, Greenberg M, Leaf P, Brown CH, Fagan A, Catalano RF, Pentz MA, Sloboda Z, Hawkins JD, Society for Prevention Research Type 2 Translational Task Force (2013). Addressing core challenges for the next generation of type 2 translation research and systems: the translation science to population impact (TSci Impact) framework. Prev Sci.

[8] Shan R, Ding J, Plante TB, Martin SS (2019). Mobile health access and use among individuals with or at risk for cardiovascular disease: 2018 Health Information National Trends Survey (HINTS). J Am Heart Assoc.

[9] Robbins R, Krebs P, Jagannathan R, Jean-Louis G, Duncan DT (2017). Health app use among US mobile phone users: analysis of trends by chronic disease status. JMIR Mhealth Uhealth.

[10] Rising CJ, Jensen RE, Moser RP, Oh A (2020). Characterizing the US population by patterns of mobile health use for health and behavioral tracking: analysis of the National Cancer Institute's health information national trends survey data. J Med Internet Res.

[11] Carroll JK, Moorhead A, Bond R, LeBlanc WG, Petrella RJ, Fiscella K (2017). Who uses mobile phone health apps and does use matter? A secondary data analytics approach. J Med Internet Res.

[12] Paré G, Leaver C, Bourget C (2018). Diffusion of the digital health self-tracking movement in Canada: results of a national survey. J Med Internet Res.

[13] Xie Z, Nacioglu A, Or C (2018). Prevalence, demographic correlates, and perceived impacts of mobile health app use amongst Chinese adults: cross-sectional survey study. JMIR Mhealth Uhealth.

[14] Banks E, Redman S, Jorm L, Armstrong B, Bauman A, Beard J, Beral V, Byles J, Corbett S, Cumming R, Harris M, Sitas F, Smith W, Taylor L, Wutzke S, Lujic S, 45 and Up Study Collaborators (2008). Cohort profile: the 45 and up study. Int J Epidemiol.

[15] Bleicher K, Summerhayes R, Baynes S, Swarbrick M, Navin Cristina T, Luc H, Dawson G, Cowle A, Dolja-Gore X, McNamara M (2022). Cohort profile update: the 45 and Up Study. Int J Epidemiol.

[16] Sax Institute (2021). Use the 45 and Up Study.

[17] (2021). Questionnaires. Sax Institute.

[18] (2021). Centre for Health Record Linkage. CHeReL.

[19] Joshy G, Korda RJ, Attia J, Liu B, Bauman AE, Banks E (2013). Body mass index and incident hospitalisation for cardiovascular disease in 158 546 participants from the 45 and Up Study. Int J Obes.

[20] Comino EJ, Tran DT, Haas M, Flack J, Jalaludin B, Jorm L, Harris MF (2013). Validating self-report of diabetes use by participants in the 45 and Up Study: a record linkage study. BMC Health Serv Res.

[21] (2017). Australian Institute of Health and Welfare. Procedures data cube under ACHI Edition 9, 2016-17.

[22] (2017). Medicines for cardiovascular disease. Australian Institute of Health and Welfare.

[23] Australian Institute of Health and Welfare (2001). Heart, stroke and vascular diseases - Australian facts 2001.

[24] (2011). Cardiovascular disease Australian facts 2011. Australian Institute of Health and Welfare.

[25] (2020). Australian Government Department of Health. Schedule of Pharmaceutical Benefits: General pharmaceutical schedule - effective 1 September.

[26] (2022). The Pharmaceutical Benefits Scheme - Browse by body system. Australian Government Department of Health.

[27] (2019). Under pressure: The squeezed middle class. OECD.

[28] (2020). Australian guidelines to reduce health risks from drinking alcohol. National Health and Medical Research Council.

[29] (2013). Australian dietary guidelines. National Health and Medical Research Council.

[30] (2021). Australia's physical activity and sedentary behaviour guidelines and the Australian 24-hour movement guidelines. Department of Health.

[31] (2021). SURE. Sax Institute.

[32] Seifert A, Vandelanotte C (2021). The use of wearables and health apps and the willingness to share self-collected data among older adults. Aging and Health Research.

[33] Bhuyan SS, Lu N, Chandak A, Kim H, Wyant D, Bhatt J, Kedia S, Chang CF (2016). Use of mobile health applications for health-seeking behavior among US adults. J Med Syst.

[34] Allen N, Sudlow C, Downey P, Peakman T, Danesh J, Elliott P, Gallacher J, Green J, Matthews P, Pell J, Sprosen T, Collins R (2012). UK Biobank: Current status and what it means for epidemiology. Health Policy and Technology.

[35] Chen Z, Chen J, Collins R, Guo Y, Peto R, Wu F, Li L, China Kadoorie Biobank (CKB) collaborative group (2011). China Kadoorie Biobank of 0.5 million people: survey methods, baseline characteristics and long-term follow-up. Int J Epidemiol.

[36] Scholtens S, Smidt N, Swertz MA, Bakker SJ, Dotinga A, Vonk JM, van Dijk F, van Zon SK, Wijmenga C, Wolffenbuttel BH, Stolk RP (2015). Cohort Profile: LifeLines, a three-generation cohort study and biobank. Int J Epidemiol.

[37] Mealing NM, Banks E, Jorm LR, Steel DG, Clements MS, Rogers KD (2010). Investigation of relative risk estimates from studies of the same population with contrasting response rates and designs. BMC Med Res Methodol.

[38] Barr M, Baker D, Gorringe M, Fritsche L (2008). NSW Population Health Survey: description of methods. New South Wales Department of Health.

[39] Wang JJJ, Bartlett M, Ryan L (2017). On the impact of nonresponse in logistic regression: application to the 45 and Up study. BMC Med Res Methodol.

[40] Ng SP, Korda R, Clements M, Latz I, Bauman A, Bambrick H, Liu B, Rogers K, Herbert N, Banks E (2011). Validity of self-reported height and weight and derived body mass index in middle-aged and elderly individuals in Australia. Aust N Z J Public Health.

[41] Foley K, Freeman T, Ward P, Lawler A, Osborne R, Fisher M (2021). Exploring access to, use of and benefits from population-oriented digital health services in Australia. Health Promot Int.

[42] Cheng C, Beauchamp A, Elsworth GR, Osborne RH (2020). Applying the electronic health literacy lens: systematic review of electronic health interventions targeted at socially disadvantaged groups. J Med Internet Res.

[43] Borg K, Boulet M, Smith L, Bragge P (2019). Digital inclusion & health communication: a rapid review of literature. Health Commun.

